# Aberrant Phase Transitions: Side Effects and Novel Therapeutic Strategies in Human Disease

**DOI:** 10.3389/fgene.2019.00173

**Published:** 2019-03-22

**Authors:** Veronica Verdile, Elisa De Paola, Maria Paola Paronetto

**Affiliations:** ^1^ University of Rome “Foro Italico”, Rome, Italy; ^2^ Laboratory of Cellular and Molecular Neurobiology, Fondazione Santa Lucia, Rome, Italy

**Keywords:** RNA-binding proteins, phase separation, RNA therapeutics, neurodegenerative disease, low-complexity domain

## Abstract

Phase separation is a physiological process occurring spontaneously when single-phase molecular complexes separate in two phases, a concentrated phase and a more diluted one. Eukaryotic cells employ phase transition strategies to promote the formation of intracellular territories not delimited by membranes with increased local RNA concentration, such as nucleolus, paraspeckles, P granules, Cajal bodies, P-bodies, and stress granules. These organelles contain both proteins and coding and non-coding RNAs and play important roles in different steps of the regulation of gene expression and in cellular signaling. Recently, it has been shown that most human RNA-binding proteins (RBPs) contain at least one low-complexity domain, called prion-like domain (PrLD), because proteins harboring them display aggregation properties like prion proteins. PrLDs support RBP function and contribute to liquid–liquid phase transitions that drive ribonucleoprotein granule assembly, but also render RBPs prone to misfolding by promoting the formation of pathological aggregates that lead to toxicity in specific cell types. Protein–protein and protein-RNA interactions within the separated phase can enhance the transition of RBPs into solid aberrant aggregates, thus causing diseases. In this review, we highlight the role of phase transition in human disease such as amyotrophic lateral sclerosis (ALS), frontotemporal dementia (FTD), and in cancer. Moreover, we discuss novel therapeutic strategies focused to control phase transitions by preventing the conversion into aberrant aggregates. In this regard, the stimulation of chaperone machinery to disassemble membrane-less organelles, the induction of pathways that could inhibit aberrant phase separation, and the development of antisense oligonucleotides (ASOs) to knockdown RNAs could be evaluated as novel therapeutic strategies for the treatment of those human diseases characterized by aberrant phase transition aggregates.

## Introduction

**Figure 1:**
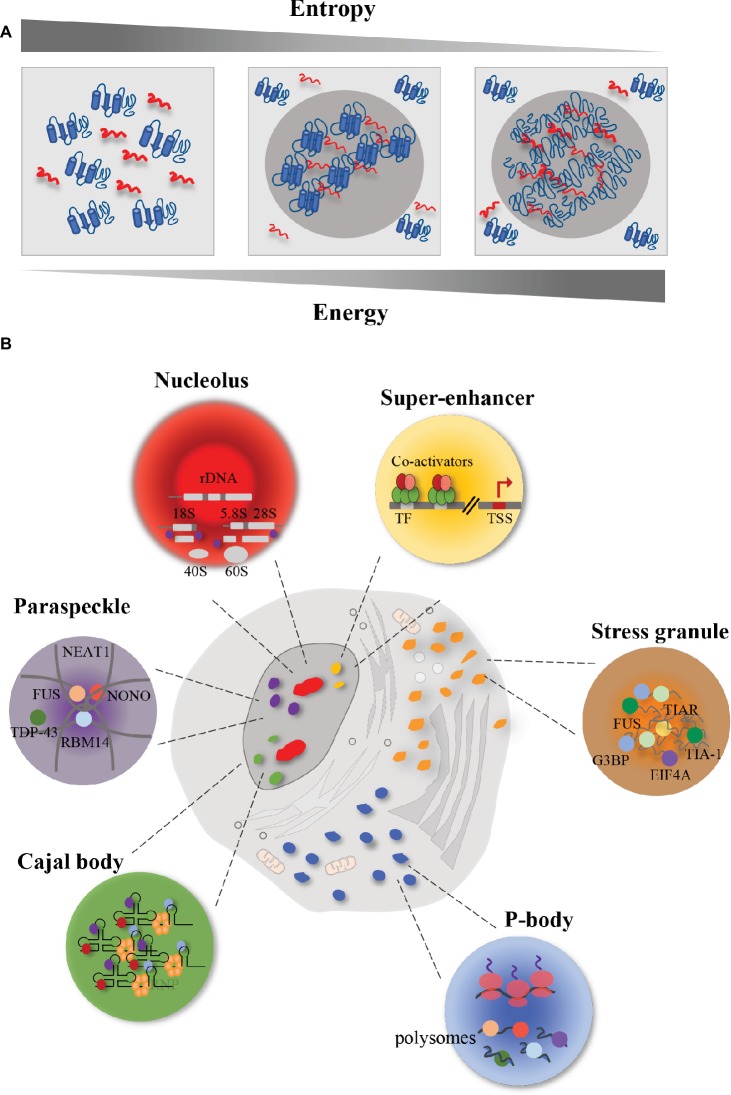
Schematic representation of membrane-less organelles (MLOs). Eukaryotic cells contain different MLOs formed by liquid–liquid phase separation. **(A)** Representation of liquid–liquid phase separation. In order to promote interaction between macromolecules and to obtain a chemical equilibrium, high energy is required with a correspondent reduction of entropy. In this condition, proteins (in blue) and RNA (in red) are prone to undergo liquid–liquid phase separation. In particular, the increase in energy and the reduction in entropy lead to a decrease in macromolecule solubility and an increase in protein-RNA interactions. The result is a higher concentrated phase, that promotes the formation of membrane-less organelles (MLOs). **(B)** Representation of membrane-less organelles (MLOs). The MLOs form sub-compartments both in the nucleus and in the cytoplasm. Nucleus is characterized by the presence of nucleoli (in red) that are involved in the ribosome biogenesis; paraspeckles (in purple), whose formation is promoted by the lncRNA *NEAT1*, that interacts with several RBPs, including FUS, in the *core,* and TDP-43 in the shell; Cajal bodies (in green) that are involved in the snRNP biogenesis; and super-enhancers (in yellow) that are clusters of master transcription factors and transcriptional co-activators involved in gene expression. In the cytoplasm, eukaryotic cells harbor P-bodies (in blue), involved in the control of mRNA translation and mRNA storage, and stress granules (in orange) whose formation, upon stress condition, requires the interaction between RNA (in grey) and proteins.

Eukaryotic cells are characterized by morphologically distinct compartments displaying multiple roles in biological processes. The complementary use of light- and electron-microscopic imaging techniques has allowed to shape eukaryotic subdomains highlighting the presence of membrane-less organelles (MLOs), including paraspeckles, nuclear speckles, Cajal bodies, stress granules (SGs), and processing bodies (P-bodies), in addition to the classical membrane-enclosed organelles (such as nuclei, mitochondria, endoplasmic reticulum, and Golgi apparatus) (Matera, 1999). These MLO compartments shape similarly, with analogous build up characteristics, but they differ in composition and sub-cellular localization. Indeed, the MLOs form sub-compartments both in the nucleus and in the cytosol (Figure 1), and contain nucleic acids and proteins necessary to accomplish their function, thus providing a spatiotemporal control of biological activities (Shin and Brangwynne, 2017). For this reason, these compartments must remain separated from cytoplasm and nucleus (Banani et al., 2017). The multiple components that concentrate within these subdomains render them a suitable interface for various cellular processes, such as transcription, RNA processing, mRNA transport, RNP assembly, ribosome biogenesis, translational repression, mRNA degradation, and intracellular signaling (Banani et al., 2017).

Several efforts have been devoted to understand the process of MLO formation and how phase separation is involved in promoting their assembly (Hyman et al., 2014). The relevance of these MLOs is demonstrated by the fact that changes in their organization are associated with disease phenotypes (Aguzzi and Altmeyer, 2016). Growing evidences suggest that these organelles are involved in the pathogenesis of neurodegenerative diseases, such as amyotrophic lateral sclerosis (ALS) and frontotemporal dementia (FTD) (Murakami et al., 2015; Rhoads et al., 2018), as well as in cancer (Aguzzi and Altmeyer, 2016; Bouchard et al., 2018).

This review explores individual MLOs, with emphasis on how they contribute to biological functions and how their dysregulation promotes the development of human disease. In this regard, we describe possible therapeutic strategies to monitor the correct formation of these compartments, and therapeutic approaches to selectively destroy aberrant MLOs.

## Phase Separation: Mechanism, Compartments, and Biological Functions

Phase separation is a physiological process by which macromolecules separate in a dense phase and in a dilute phase within cells, thus allowing the formation of distinct chemical environments (Shin and Brangwynne, 2017). In order to promote interaction between macromolecules, rather than between macromolecules and solvent, and to obtain a chemical equilibrium among the compartments just formed, high energy is required (Johansson et al., 1998), with a correspondent reduction of entropy (Figure 1A). The result is a higher concentrated phase, where the proteins and DNA/RNA cluster ~ 10–100-fold more (Li et al., 2012), and a lower concentrated phase (Banani et al., 2017). In the phase separation process, macromolecule solubility is decreased (Alberti, 2017). Liquid–liquid phase separation (LLPS) occurs spontaneously in eukaryotic cells when a critical concentration or temperature threshold is exceeded, thus forming sub-compartments in a reversible fashion (Hyman et al., 2014; Shin and Brangwynne, 2017). The assembled MLOs must remain separated by the surrounding environment, both in the nucleus and in the cytoplasm. These organelles in turn support the transport of molecules in and out themselves, thus allowing chemical reactions inward (Hyman et al., 2014).

To obtain liquid–liquid demixing, several conditions are required, such as electrostatic and hydrophobic interactions, achieved by the presence of low-complexity sequences, and intrinsically disordered protein regions (IDRs) (Nott et al., 2015), that are involved in protein–protein (Lee et al., 2015), and protein-RNA interactions (Allain et al., 2000). RNA can initiate by itself phase separation *via* protein-RNA (Molliex et al., 2015) and RNA–RNA interactions (Jain and Vale, 2017; Van Treeck et al., 2018) and serves as a molecular seed that triggers liquid demixing (Molliex et al., 2015).

Eukaryotic cells harbor MLOs both in the nucleus and in the cytoplasm. The first MLO identified was the **P granule** of *Caenorhabditis elegans* embryos, involved in cytoplasmic polar partitioning (Strome and Wood, 1983). P granules have been defined as a liquid-like compartment. Indeed, they display a spherical morphology due to surface tension. They can fuse together with other P granules and be deformed by flows, then rapidly rearranging (Brangwynne et al., 2009). P granules contain mRNAs and RNA helicases and play a key role in the post-transcriptional regulation of mRNA in the germ cells (Voronina et al., 2011).

In the cytoplasm, eukaryotic cells can form processing bodies (P-bodies) and stress granules (Figure 1B). **P-bodies** are cytoplasmic ribonucleoprotein (RNP) granules (Franks and Lykke-Andersen, 2008). Fluorescence microscopy has shown that proteins and RNAs shuttle between cytoplasm and P-bodies and fuse, showing liquid-like properties (Kedersha et al., 2005). These RNP granules play a role in post-transcriptional regulation, by controlling mRNA translation and degradation (Parker and Sheth, 2007). Indeed, their assembly depends on the loading of mRNAs into polysomes. When mRNAs are associated with ribosomes, P-bodies decrease in abundance and size (Sheth and Parker, 2003; Teixeira et al., 2005); whereas when mRNAs dissociate from ribosomes, as a result of translation inhibition, P-bodies increase in dimensions (Teixeira et al., 2005; Koritzinsky et al., 2006). Indeed, mRNA decay can occur also in absence of P-bodies (Eulalio et al., 2007), thus assuming that P-bodies act by segregating mRNAs rather than degrading them (Parker and Sheth, 2007).

In addition to P-bodies, cytoplasm of eukaryotic cells harbors other types of RNA granules, such as **stress granules (SGs)** (Collier and Schlesinger, 1986; Arrigo et al., 1988). Fluorescence microscopy (Jain et al., 2016) and electron-dense regions’ micrographs (Souquere et al., 2009) revealed that SGs have a highly concentrated *core* made up by proteins and mRNA, and a surrounding structure which is less dense and more dynamic (Protter and Parker, 2016). Phase separation is extremely sensitive to changes in chemical conditions, thus playing an important role in stress adaptation. In fact, stress is a transient phenomenon and SGs are transient structures rapidly disassembling upon removal of the stress condition. For instance, after removal of the adverse condition, SGs disassemble, protein synthesis is reactivated after the translation inhibition induced by the stress, and RBPs can either go back to the nucleus or remain in the cytoplasm to carry out their functions (Gilks et al., 2004; Panas et al., 2016).

The formation of these bodies occurs when stress conditions block translation initiation, by phosphorylating eIF2α or inactivating eIF4A (Mokas et al., 2009). In general, this block induces sudden increase in non-polysomal mRNAs, masked by translating ribosome, that is free to interact with RBPs, such as TIA-1,TIA-R, and G3BP (Kedersha et al., 1999; Tourrière et al., 2003). These proteins interact with other proteins through specific domains, thus promoting SG formation (Protter and Parker, 2016). Phase transition can be promoted and largely affected by post-translational modifications of SG-associated protein, such as methylation, phosphorylation, and glycosylation, that alter protein–protein interaction (Tourrière et al., 2003; Ohn et al., 2008; Nott et al., 2015).

In general, SGs help cells to respond to adverse conditions, such as oxidative stress, heat shock, and DNA damage (Kedersha and Anderson, 2007; White and Lloyd, 2012). Upon DNA damage, Moutaoufik and colleagues observed that UV-induced SGs were smaller and less numerous than the SGs induced by other stressors, such as arsenite or heat treatment (Moutaoufik et al., 2014). The cellular response to UVC irradiation involves several steps that allow cells to identify the damage and to repair the DNA. These processes include the surveillance of genome integrity, the recognition of damaged DNA, the activation of the DNA repair program, including cell signaling events and cell cycle arrest, thus allowing cells to repair the DNA before resuming proliferation (Polo and Jackson, 2011). Indeed, it has been shown that upon UV-induced DNA damage, SG assembly occurs in a cell cycle-dependent fashion (Pothof et al., 2009), with cyclin A-positive S phase cells and γH2AX-positive cells negative for SG-specific staining (Pothof et al., 2009). These studies demonstrate that SG formation occurs only in a time window of G2-M transition or after exit from mitosis. In fact, as cells prepare for cell division, most MLOs disassemble and then start to reassemble during the late stages of cytokinesis (Andrade et al., 1993; Hernandez-Verdun et al., 2002; Fox et al., 2005; Aizer et al., 2013). In particular, the kinase activity of DYRK3 plays an essential role during mitosis to prevent the formation of aberrant LLPS condensates composed by nuclear and cytoplasmic proteins and RNA, by keeping the condensation threshold of its substrates high (Rai et al., 2018).

During stress, fluctuations in cytosolic pH can promote widespread condensate formation and a core group of nucleating RBPs is sufficient to initiate formation of the stress granule. For instance, in budding yeast, cells can enter into a quiescent state upon removal of nutrients that causes a shift in the cytosolic pH from 7.4 down to ~6.0. This increase in proton concentration triggers a phase transition; as soon as conditions improve, yeast fluidize the cytoplasm by using proton pumps and neutralizing the pH, thus restoring normal conditions (Munder et al., 2016).

Collectively, phase separation offers a suitable architecture to regulate and compartmentalize biochemical processes inside cells.

Several MLOs form within the cell nucleus (Figure 1B). A typical example of MLO with liquid-like properties is the **nucleolus** that forms around ribosomal DNA loci in the cell nucleus (Shaw and Jordan, 1995; Brangwynne et al., 2011). Nucleoli are RNA-protein compartments displaying a key role in the ribosome biogenesis (Andersen et al., 2002). Taking advantage of the electron microscopy, it has been shown that this process occurs in three distinct sub-regions of the nucleolus, formed as a result of LLPS (Boisvert et al., 2007). The transcription of rDNA starts in the fibrillar centers sub-region, that is enriched in RNA polymerase I (RNAPI). Then, this process continues in the dense fibrillar components sub-region, where also processing and modification of pre-rRNA transcripts occur. The assembly of the ribosome is accomplished in the granular components, enriched in proteins (Boisvert et al., 2007). This organization into sub-compartments resembling a multi-layer structure has been found also in other liquid-like MLOs in the cell nucleus, including paraspeckles (Feric et al., 2016).

**Paraspeckles** are nuclear bodies involved in the control of gene expression and DNA repair, and characterized by the presence of RBPs, including the *Drosophila* behavior/human splicing (DBHS) family of splicing proteins (the paraspeckle protein 1 PSPC1, RBM14, and NONO), FUS and TDP-43 proteins (Fox et al., 2002). It has been shown that the formation of paraspeckles is driven by RNA, in particular by the long non-coding RNA (lncRNA) *NEAT1* (Souquere et al., 2010). Knockdown of *NEAT1* leads to the disintegration of paraspeckles (Clemson et al., 2009). Indeed, paraspeckles form their *core* around the central part of *NEAT1*, whereas the surrounding structures (the shell and the patch) form around its 5′ and 3′ ends (Souquere et al., 2010). Interestingly, it has been shown that FUS localizes in the *core*, whereas TDP-43 concentrates in the shell (Hennig et al., 2015). This different localization reflects distinct roles displayed by the two RBPs in paraspeckles formation. In *Fus−/−* mice, *Neat1* accumulated at its transcription sites but did not form the *core-*shell structure; moreover, it was found diffused throughout the nucleoplasm (West et al., 2016). Interestingly, in *Fus−/−* mice, the *core* group proteins SFPQ, NONO, and PSPC1 accumulated at the *Neat1* transcription sites, indicating that FUS protein was not essential for their association with *Neat1*. On the contrary, the patch protein BRG1 and RBM14 were not enriched at *Neat1* transcription sites, indicating an essential role for FUS in stabilizing the interaction of these proteins with nascent *Neat1* transcripts (West et al., 2016). On the other hand, TDP-43 downregulation induced the accumulation of *NEAT1* transcripts and the formation of paraspeckles (Shelkovnikova et al., 2018). This accumulation was probably due to the protective role displayed by paraspeckles against defective miRNA pathway, caused by TDP-43 depletion (Shelkovnikova et al., 2018).

Electron microscopy has allowed the identification of other MLOs in the nucleus, such as the **Cajal body** (Gall et al., 1999). Cajal bodies show a coiled structure and form on active snRNA loci (Frey and Matera, 2001). They share the same properties of other MLOs (Handwerger et al., 2003; Kato et al., 2012). Their assembly is initiated by small nuclear RNAs (snRNAs), including small nucleolar RNAs (snoRNAs) and small Cajal body-specific RNAs (scaRNAs), that interact with RBPs, which in turn recruit other proteins (Machyna et al., 2013). An essential aggregation factor of these structures is the protein coilin, which through its multi-modular domains gathers RBPs and RNAs, leading to the formation of Cajal bodies (Machyna et al., 2014). These bodies maintain structural integrity during interphase (Carmo-Fonseca et al., 1993), and are implicated in the small nuclear ribonucleoprotein (snRNP) biogenesis, spliceosome formation, telomere maturation, and maintenance (Machyna et al., 2013).

In addition to MLOs, a phase separation model has been recently proposed to explain basic mechanisms of the transcriptional regulation, such as **super-enhancers** (Hnisz et al., 2017; Sabari et al., 2018). Super-enhancers are clusters of transcriptional enhancers assembled by simultaneous binding of master transcription factors, transcriptional co-activators, RNAPII and RNA, that drive the expression of genes involved in defining cell identity (Whyte et al., 2013). Many molecules bound at enhancer-regions can undergo reversible chemical modifications (e.g., acetylation, phosphorylation, methylation) at multiple sites. Upon such modifications, these molecules change their interactome, thus promoting changes in the overall charge and in the affinities of the interacting molecules, and obtaining a high-density assembly of biomolecules at active sites (Hnisz et al., 2017; Sabari et al., 2018). In this way, super-enhancers are susceptible to perturbation and their activity is fine-tuned by internal and external cues. During the process of tumor pathogenesis, chromosomal translocation or overexpression of oncogenic transcription factors favors the formation of super-enhancer at sites of oncogenes (Hanahan and Weinberg, 2011), thus driving aberrant gene expression programs. Phase separation can also be promoted by proteins involved in the proteasome-degradation pathway (Li et al., 2014). In the case of the tumor suppressor SPOP (speckle-type POZ protein), involved in ubiquitination and proteasomal degradation of substrates (Li et al., 2014), target proteins drive SPOP-mediated separation process, and when cancer-associated mutations of *SPOP* gene occur, substrate binding and phase separation are displaced (Bouchard et al., 2018). SPOP localizes in various nuclear bodies including speckles and DNA-damage *loci* (Nagai et al., 1997; Marzahn et al., 2016). Cancer mutations in SPOP negatively regulate the LLPS process between SPOP and substrates and prevent their ubiquitination, leading to upregulation of these proteins and impaired proteostasis (Bouchard et al., 2018).

Thus, phase transitions in MLOs and at super-enhancers allow the accomplishment of gene expression programs both in healthy and diseased cellular states.

## Phase Separation Promoted by Low-Complexity RNA-Binding Proteins

A well-defined protein structure is essential to accomplish protein functions within the cell. However, many protein portions lack a well-defined structure still remaining functional. These segments are indicated as intrinsically disordered regions (IDRs) and proteins harboring them are named intrinsically disordered proteins (IDPs) (Li et al., 2012). Unlike globular proteins, IDPs use only a subset of the 20 amino acids, with low content of hydrophobic amino acids. To achieve phase separation, the exact amino acid sequence of IDPs is not important, while the overall composition and charge pattern are extremely relevant. Initially considered as passive segments linking structured domains, IDRs actively participate in different cellular functions, and their activity is fine-tuned by post-translational modifications (Iakoucheva et al., 2004; Collins et al., 2008).

In order to obtain the separation from nucleoplasm and cytoplasm, MLOs contain IDPs harboring low-sequence complexity domains (LCDs) (Gilks et al., 2004; Han et al., 2012; Kato et al., 2012; Toretsky and Wright, 2014). These domains are also present in yeast prion proteins (Alberti et al., 2009), from which the term “prion-like” is derived. Prions are infectious protein conformers capable of self-replication (Shorter and Lindquist, 2005). Prion-like domains (PrLDs) are a type of LCD with a tendency to self-assemble and form aggregates. Their ability to form amyloid is dependent on the PrLD rich in glycine and uncharged polar amino acids to reduce the solubility of the proteins (Alberti et al., 2009). Deletion of the prion domain precludes the formation of the prion conformer (Masison et al., 1997), while the addition of this region to a given protein is sufficient to confer prion behavior (Li and Lindquist, 2000). Remarkably, mutations in PrLD-containing proteins cause devastating protein-misfolding diseases, characterized by the formation of solid aggregates (Kim et al., 2013; Li et al., 2013; Ramaswami et al., 2013).

Proteins involved in RNA processing display high phase separation propensities (Vernon et al., 2018). Indeed, as mentioned above, MLOs contain several RBPs harboring PrLDs. Remarkably, of the 240 human proteins harboring predicted PrLDs, 72 (30%) are involved in RNA metabolism (March et al., 2016). These proteins are recently emerging in the pathology and genetics of human neurodegenerative diseases (King et al., 2012).

ATXN1 and ATXN2 were the first RBPs with a putative PrLD to be linked to the pathogenesis of neurodegenerative diseases, causing, respectively, the type 1 and type 2 spinocerebellar ataxia (Banfi et al., 1994; Lorenzetti et al., 1997), a neurodegenerative disorder characterized by an expansion of a trinucleotide CAG repeat within the coding region of the *SCA1* and *SCA2* genes (Banfi et al., 1994; Lorenzetti et al., 1997). In physiological conditions, ATXN1 is located both in the nucleus and in the cytoplasm (Servadio et al., 1995), and is able to shuttle between these two compartments. However, ATXN1 dynamics is altered by the expansion; in fact, while mutated ATXN1 is still able to enter the nucleus, its ability to be transported back into the cytoplasm is dramatically reduced (Irwin et al., 2005). On the contrary, ATXN2 is mainly localized into the cytoplasm, associated to translating polysomes or into stress granules and P-bodies, where it is involved in the regulation of translation, mRNA storage, or degradation (Orr, 2012).

**Figure 2:**
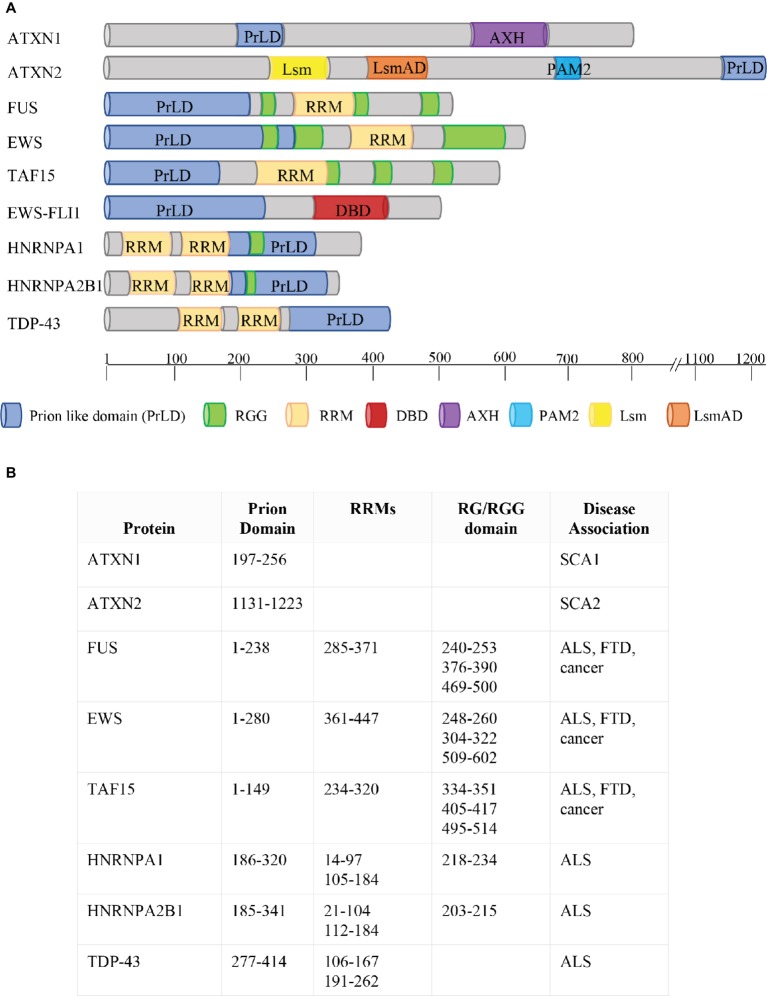
Phase Separation by RBPs. **(A)** Schematic representation of protein domains of RBPs involved in FTD, ALS, and cancer. ATXN1 and ATXN2 contain two predicted prion-like domains (ATXN1: aa197–256; ATXN2: aa1131–1,223; March et al., 2016). The FET proteins FUS, EWS, and TAF15 combine two types of low-complexity domain (LCD): prion-like domains (PrLDs) and RGG-rich domains. The two types of LCDs cooperate to drive the dynamic phase separation. FET proteins are frequently translocated in human cancers, with the resulting fusion proteins (e.g. EWS-FLI1) lacking either significant parts of the RGG-rich LCD or the RNA recognition motif (RRM) but containing the DNA-binding domain (DBD) of an ETS transcription factor (e.g. FLI1). HNRNPA1 and HNRNPA2 combine two RRM motifs with a PrLD, together with an RG domain. TDP43 contains two RRM motifs and a PrLD. Protein domains are indicated in different colors (see legend). AXH = Ataxin-1 and HMG-box protein domain; Lsm = Like RNA splicing domain Sm1 and Sm2; LsmAD = Like-Sm-associated domain; PAM2 = poly (A)-binding protein interacting motif. The location of each protein domain and the association with human pathologies are indicated in **(B)**. SCA1 = spinocerebellar ataxia type 1; SCA2 = spinocerebellar ataxia type 2; ALS = Amyotrophic Lateral Sclerosis; FTD = Frontotemporal Dementia.

Almost 10 years later, the transactive response (TAR) DNA-binding protein 43 kDa (TDP-43) was associated with a neurodegenerative disease (Arai et al., 2006; Neumann et al., 2006). TDP-43 is a RBP containing a PrLD (amino acids 277–414, Figure 2), is localized in the cell nucleus, but shuttles to the cytoplasm displaying roles in transcriptional and post-transcriptional RNA processing (Buratti and Baralle, 2008, 2010). TDP-43 misfolding has been connected to the pathology of ALS and frontotemporal lobar degeneration with ubiquitin-positive inclusions (FTLD-U) (Neumann et al., 2006; Chen-Plotkin et al., 2010; Da Cruz and Cleveland, 2011). In these disorders, TDP-43 displays a cytoplasmic localization and forms aggregates; moreover, it is depleted from the nucleus of diseased neurons (Chen-Plotkin et al., 2010; Da Cruz and Cleveland, 2011).

The PrLD of FUS harbors amino acids 1–238 (Figure 2). Like TDP-43, FUS is mainly localized in the nucleus, but shuttles to accomplish functions in transcriptional and post-transcriptional regulation, RNA processing, and miRNA biogenesis (Bertolotti et al., 1996; Zinszner et al., 1997; Paronetto, 2013; Svetoni et al., 2016). Mutations in FUS cause familial ALS (Kwiatkowski et al., 2009; Vance et al., 2009; Da Cruz and Cleveland, 2011) and FTLD-U (Neumann et al., 2009; Mackenzie et al., 2010; Da Cruz and Cleveland, 2011; Drepper and Sendtner, 2011; Svetoni et al., 2016). In these pathologies, FUS is localized in cytoplasmic aggregates of the degenerating neurons (Mackenzie et al., 2010). FUS belongs to the FET family of RBPs, composed by FUS/TLS, EWS, and TAF15, and involved in multiple steps of RNA metabolism (Svetoni et al., 2016). FET proteins share a common domain architecture (Paronetto, 2013). Upon inhibition of transcription (Zinszner et al., 1997) or DNA damage (Paronetto et al., 2011), they translocate into the nucleoli forming dense nuclease-resistant aggregates. In Ewing sarcoma, the replacement of the RNA-binding domains of FET proteins with an ETS transcription factor due to chromosomal translocations alters their nucleic acid-binding affinities and activities, thus causing activation of a transcriptional program leading to cancer transformation (Paronetto, 2013). As mentioned, in neurodegenerative diseases, point mutations in the genes encoding FET proteins affect their localization and aggregation propensity, strongly supporting the hypothesis that phase transition contributes to the development of pathological conditions (Svetoni et al., 2016).

In 2011, mutations in the gene encoding TAF15 have been identified in ALS and FTLD-U patients (Couthouis et al., 2011; Neumann et al., 2011; Ticozzi et al., 2011). Interestingly, both TAF15 and EWS harbor a prominent N-terminal PrLD (amino acids 1–149 in TAF15; amino acids 1–280 in EWS; Figure 2) enriched in glutamine residues, which might enhance the formation of toxic oligomeric structures (Halfmann et al., 2011).

hnRNPA1 and hnRNPA2 are prototypical hnRNPs formed by two folded RNA recognition motifs (RRMs) in the N-terminal part of the protein and a PrLD in the C-terminal (amino acids 185–341 in hnRNPA2; amino acids 186–320 in hnRNPA1; Figure 2) (Kim et al., 2013), involved in the interaction with TDP-43 (Buratti et al., 2005). Missense mutations in the PrLD of hnRNPA1 and hnRNPA2 have been identified in ALS patients (Kim et al., 2013). HnRNPA2 and hnRNPA1 are prone to fibrillization, which is enhanced by disease-causing mutations (Kim et al., 2013). Notably, hnRNPA2B1 and hnRNPA1 mutations have been identified in families presenting multisystem proteinopathy (MSP) (Benatar et al., 2013; Le Ber et al., 2014), a rare complex phenotype which involves perturbation of SG dynamics and autophagic protein degradation, affecting muscle, brain, and bone (Benatar et al., 2013). MSP phenotype associates different disorders, such as frontotemporal lobar degeneration (FTLD), Paget disease of bone (PDB), inclusion body myopathy (IBM), and ALS (Benatar et al., 2013).

Aromatic residues play important roles in IDR interactions; they mediate short-range, aromatic interactions and promote LLPS, whereas hydrophilic residues control the solubility of IDRs and counteract LLPS (Kato et al., 2012; Xiang et al., 2015). For instance, tyrosine mutations block recruitment of hnRNPA2 and FUS IDRs into phase-separated liquids as well as into RNA granules (Kato et al., 2012; Xiang et al., 2015). The numerous tyrosine residues in FUS contribute to LLPS, and their recruitment into LLPS is controlled by phosphorylation (Lin et al., 2017). In fact, phosphorylation enables rapid transitions within the IDRs and controls the assembly/disassembly of the RNP granules (Lin et al., 2017).

Electrostatically driven phase separation can be also promoted by the interaction of arginine/glycine-rich domains with RNA. The RGG/RG repeats, often present in RBPs, usually occur in LCD. Compared to sequences from ordered proteins, these IDRs typically exhibit high levels of a subset of specific amino acids, that promote phase separation on their own. In particular, the polyvalent interactions between arginines and RNA achieve the phase separation process (Romero et al., 2001). RGG-containing regions mediate RNA binding (Kiledjian and Dreyfuss, 1992) and can be methylated by PRMTs (Bedford and Richard, 2005; Bedford and Clarke, 2009). Methylation is a post-transcriptional modification that negatively influences the capability of RBPs to bind RNA. For instance, methylation of the RGG/RG motif of FMRP reduces its affinity for RNA (Stetler et al., 2006) and its recruitment on polysomes (Blackwell et al., 2010). Moreover, methylarginines within the RG motifs of the RBP Sam68 negatively influence its affinity for the SH3 domains (Bedford et al., 2000). Arginine methylation also decreases the interaction of FUS with transportin, thus affecting its nuclear import (Dammer et al., 2012). To this regard, methylation of the RGG/RG motifs affects the localization of FUS proteins harboring ALS-linked mutations (Tradewell et al., 2012). Notably, mutations in the RGG/RG domains of FUS have been identified in familial cases of ALS (Kwiatkowski et al., 2009; Hoell et al., 2011).

The presence of RGG/RG motifs within a given protein can be regulated by alternative splicing choices (Blackwell and Ceman, 2011). For instance, isoform 12 of *FMR1* excludes exons 12 and 14, thus leading to a truncated FMRP isoform defective of the RGG domain encoded by exon 15. This FMRP isoform displays reduced localization to dendritic RNA granules (Mazroui et al., 2003; Blackwell and Ceman, 2011).

Chromosomal translocations between the *EWSR1* gene and genes encoding ETS transcription factors can cause aggressive pediatric tumors designated as Ewing sarcomas (Araya et al., 2005; Paronetto, 2013). The translocations result in chimeric proteins harboring the N-terminal activation domain of EWS, comprehending the PrLD, fused to the DNA-binding domain of an ETS transcription factor, but lacking the C-terminal domain of EWS containing the RNA-binding regions (the RGG motifs and the RRM motif; Figure 2). Notably, the RGG/RG motifs of EWS display an inhibitory activity toward the DNA activation domain, thus decreasing its oncogenic potential (Li and Lee, 2000). Interestingly, the translocated PrLD deriving from EWS promotes the aggregation of EWS-FLI1 in foci (Boulay et al., 2017). The presence of aromatic residues affects the aggregation propensity of EWS-FLI1. In fact, replacement of the 37 tyrosine residues with serines removes its ability to form aggregates, and this tyrosine-replaced variant is unable to assemble active enhancers (Boulay et al., 2017). Since RNA helicases are extensively involved in phase separation dynamics (Nott et al., 2015), it is possible that the interaction between EWS-FLI1 and the DNA–RNA helicase DHX9 plays an essential role in the formation of these aggregates; thus, blocking this interaction could be a strategy to limit EWS-FLI1 oncogenic potential (Fidaleo et al., 2016). Since the ability of the EWS-FLI1 PrLD to phase separate is closely linked to its oncogenic activity, preventing or reverting phase separation properties could have therapeutic utility in Ewing sarcoma.

## Therapeutic Approaches

**Figure 3:**
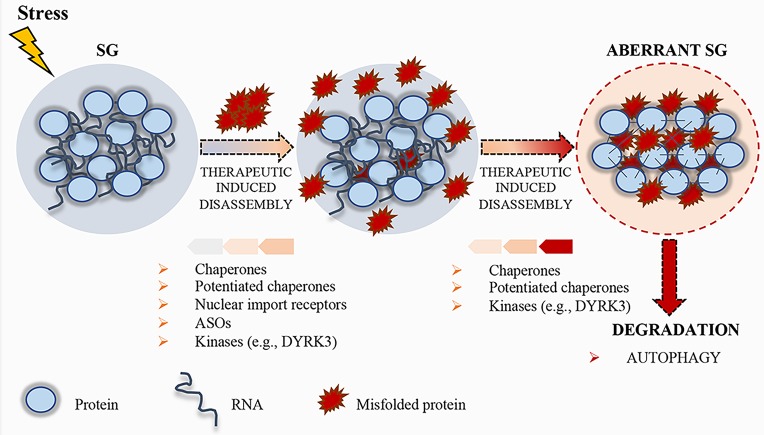
Schematic representation of stress granule (SG) dynamics. Upon stress condition, SGs assemble; RNA and proteins accumulate in these granules until the stress persists. After removal of the stress condition, SGs disassemble, protein synthesis is reactivated after the translation inhibition induced by the stress, and RBPs can either go back to the nucleus or remain in the cytoplasm, where they carry out their normal functions. Misfolded proteins can accumulate in SGs, thus altering their physical properties. To prevent the accumulation of misfolded proteins, therapeutic strategies, such as chaperones, potentiated chaperones, protein kinases (e.g., DYRK3), nuclear import receptors, and ASOs, can be engineered to induce SG disassembly. If the disassembly does not occur, SGs become aberrant and can promote the pathogenesis of neurodegenerative disorders. Finally, aberrant SGs can undergo disassembly by chaperones, potentiated chaperones, or kinases, and, if not repaired, they undergo degradation by autophagy.

Phase separation displays a crucial role in neurodegenerative disorders. Several proteins involved in neurodegenerative diseases are components of MLOs and dysregulation in the formation or conservation of these components leads to pathological aggregates (Li et al., 2013; Ramaswami et al., 2013). Therefore, the development of novel therapeutic strategies to control cellular phase transition could be instrumental for the treatment of those human diseases characterized by aberrant aggregates (Figure 3).

In addition to RBPs, several ALS mutations have been identified in genes encoding members of the protein quality control system (PQC), including chaperones, components of the ubiquitin/proteasome, or autophagolysosomal system (Robberecht and Philips, 2013; Capponi et al., 2016; Alberti et al., 2017). These systems display a crucial role in the control of protein aggregation. Chaperones recognize SGs containing misfolded aggregated proteins (Mateju et al., 2017). When a specific chaperone mechanism is compromised, misfolded proteins and defective ribosomal products accumulate into SGs, thus altering SG dynamics and causing defects in SG disassembly (Ganassi et al., 2016). Ganassi and collaborators identified the HSPB8-BAG3-HSP70 chaperone complex as a key regulator of SG surveillance. The incidence of aberrant defective ribosomal products-containing SGs in normal conditions is very low, suggesting that the PQC is highly efficient in preventing aberrant SG formation (Ganassi et al., 2016). On the same line, Mateju and collaborators demonstrated that SGs containing ALS-associated SOD1 aggregates engage increased number of chaperones, including HSP27 and HSP70, suggesting their specific enrollment to avoid aberrant SGs (Mateju et al., 2017). Treatment with a chemical inhibitor of HSP70 increases the number of SGs containing misfolded proteins, suggesting that HSP70 hampers the accumulation of misfolded proteins and facilitates a rapid disassembly of SGs in the recovery phase (Mateju et al., 2017). Thus, surveillance of SGs by chaperones is critical for the maintenance of their normal composition and dynamics, and the stimulation of the chaperone machinery could be a useful target to disassemble MLOs. In this context, the development of potentiated chaperones could also be a suitable approach to optimize therapeutic efficacy against neurodegenerative diseases (Shorter, 2008; Jackrel et al., 2014; Yasuda et al., 2017). To this regard, Jackrel and collaborators were able to potentiate HSP104 variants from yeast (Jackrel et al., 2014; Jackrel and Shorter, 2014). In particular, the developed enhanced chaperone was able to revert TDP-43 and FUS aggregation, thus suppressing their toxicity and eliminating protein aggregates in yeast (Jackrel et al., 2014; Jackrel and Shorter, 2014). Moreover, Yasuda and collaborators demonstrated for the first time that engineered HSP104 variants are able to dissolve cytoplasmic ALS-linked FUS aggregates in mammalian cells (Yasuda et al., 2017).

Another approach to stimulate the chaperone machinery is to develop drugs able to upregulate the expression levels of heat shock proteins (HSPs). Arimoclomol (BRX-345) is a hydroxylamine derivative that facilitates the formation of chaperone molecules by enhancing the expression of heat shock genes (Vigh et al., 1997, Kieran et al., 2004). Arimoclomol treatment in *SOD1G93A* mice was shown to upregulate HSP70 and HSP90 expression, leading to a significant delay in the progression of the disease (Kieran et al., 2004). Remarkably, a phase II/III randomized, double-blind, placebo-controlled clinical trial is currently underway in familial SOD1-ALS patients (NCT00706147). Furthermore, arimoclomol treatment has been shown to induce a reduction in the pathological markers *in vitro* and amelioration of pathological and functional deficits *in vivo* of the sporadic inclusion body myositis (sIBM), a severe myopathy characterized by protein dys-homeostasis (Ahmed et al., 2016).

Since the disruption of the ubiquitin-proteasome-system and autophagy are central events in ALS, current research is now focusing on the development of drugs able to upregulate the signaling pathways involved in PQC (Barmada et al., 2014). For instance, new compounds stimulating autophagy are able to improve TDP-43 clearance and localization, thus mitigating neurodegeneration (Barmada et al., 2014). Stimulation of autophagy also enhances survival of human-induced pluripotent stem cells (iPSC)-derived neurons and astrocytes from patients with familial ALS (Barmada et al., 2014). Furthermore, great effort has recently been devoted to improving HSPB8 function and to promote the autophagy-mediated removal of misfolded mutant SOD1 and TDP-43 fragments from ALS motor neurons. To this regard, colchicine treatment enhances the expression of HSPB8 and of several autophagy players, while blocking TDP-43 accumulation in neurons (Rusmini et al., 2017). Based on these premises, a phase II randomized, double-blind, placebo-controlled, multicenter clinical trial has been activated to test the efficacy of colchicine in ALS (NCT03693781).

Recently, Guo and collaborators showed the relevance of nuclear-import receptors (NIRs) in the disaggregation of disease-linked RBPs with a nuclear localization signal (NLS) (Guo et al., 2018). The binding of Karyopherin-β2, also named transportin-1, to the PY-NLSs is sufficient to revert FUS, TAF15, EWS, hnRNPA1, and hnRNPA2 fibrillization, while Importin-α, in complex with Karyopherin-β1, reverts TDP-43 fibrillization (Guo et al., 2018). Karyopherin-β2 avoids the aberrant accumulation of RBPs containing PY-NLSs into the SGs and re-establishes proper RBP nuclear localization and function, thus rescuing the degeneration caused by mutated FUS and hnRNPA2 (Guo et al., 2018). Thus, NIRs might contribute to the setup of novel therapeutic strategies to restore RBP homeostasis and moderate neurodegeneration.

Recent evidences highlight the role of specific kinases in the regulation of the dissolution of SGs and other MLOs. For instance, during recovery from stressful conditions, the kinase activity of DYRK3 is required for SG dissolution and restoration of mTORC1 activity (Wippich et al., 2013). DYRK3 binds MLO proteins and phosphorylates their LCDs (Wippich et al., 2013), thus affecting the electrostatic properties of these domains and the condensation threshold of the proteins harboring them (Rai et al., 2018). Importantly, DYRK3 has been shown to act as a dissolvase of liquid-unmixed compartments (Rai et al., 2018). In fact, upon overexpression, recombinant DYRK3 was able to dissolve MLOs both in the nucleus and in the cytoplasm in a kinase-activity-dependent fashion (Rai et al., 2018). Similar to DYRK3, Casein kinase 2 (CK2) was recently found to cause SG disassembly *via* phosphorylation of the SG nucleating protein G3BP1 (Reineke et al., 2017). Thus, identification of kinases, such as DYRK3 and CK2, able to modulate MLO dissolution and/or drugs that regulate their functions may represent another interesting therapeutic approach (Figure 3).

Finally, a suitable approach to downregulate key regulators involved in aberrant phase transitions is the use of antisense oligonucleotides (ASOs). ASOs can be used to target pathological proteins in different mouse models (Schoch and Miller, 2017). In case of essential proteins, ASO-strategy could be engineered to target non-essential partners, involved in the regulation of phase transition (Boeynaems et al., 2018). This is the case of TDP-43 and ataxin-2. Ataxin-2 is an RBP with multiple roles in RNA metabolism, such as regulation of SG assembly (Elden et al., 2010; Kaehler et al., 2012). Reduction of ataxin-2 by ASOs affects SG dynamics and decreases recruitment of TDP-43 to SGs (Becker et al., 2017). A single administration of ASOs targeting ataxin-2 into the central nervous system is sufficient to increase the lifespan and improve motor function of TDP-43 transgenic mice (Becker et al., 2017). Since alterations in TDP-43 have been found in 97% of ALS cases and about 50% of FTD cases (Ling et al., 2013), the reduction of ataxin-2 could be used as therapeutic strategy for ALS and FTD treatment (Wils et al., 2010).

To conclude, the stimulation of chaperone machinery, the induction of pathways triggering PQC, and the development of ASOs could be exploited to set up novel therapeutic approaches for the treatment of those human diseases characterized by aberrant phase transition aggregates.

## Concluding Remarks

Membrane-less subcellular organization plays a pivotal role in cellular homeostasis. LCDs, including PrLDs and RGG domains, behave as general scaffolds in assisting MLO’s activity and mediating the dynamics of RNP granules. Persistence of RNP granules caused by either failure of granule removal, mutated PrLD-containing RBPs, or granule-associated misfolded proteins can lead to pathological protein aggregates, that contribute, at least in part, to the pathogenesis of neurodegenerative diseases. At the same time, chromosomal translocation can promote the formation of aberrant chimeric proteins formed by PrLD fused to transcription factors; these translocated PrLDs promote phase separation and activate transcriptional programs driving transformation (Boulay et al., 2017). On the other hand, disruption of membrane-less organization by mutations in the tumor suppressor SPOP can cause solid tumors (Bouchard et al., 2018).

Given the relevance to human health, determining how LCD proteins organize cellular compartments could be instrumental to expand our understanding of compartment formation, thus providing significant insight into neurodegenerative pathologies and cancer. Recent studies document how pernicious misfolding can be reversed by protein disaggregases (Shorter, 2008; Torrente and Shorter, 2013; Jackrel and Shorter, 2015), opening the path to novel promising therapeutic applications both in cancer treatment and in the cure of neurodegenerative disease. Yet, the exact MLO dynamics has not been completely unraveled, neither in physiological or pathological conditions.

## Author Contributions

VV, EDP and MPP analyzed the literature and wrote the manuscript.

### Conflict of Interest Statement

The authors declare that the research was conducted in the absence of any commercial or financial relationships that could be construed as a potential conflict of interest.

## References

[ref1] Aguzzi A. Altmeyer M. (2016). Phase separation: linking cellular compartmentalization to disease. Trends Cell Biol. 26, 547–558. 10.1016/j.tcb.2016.03.004, PMID: 27051975

[ref2] Ahmed M. Machado P. M. Miller A. Spicer C. Herbelin L. He J.. (2016). Targeting protein homeostasis in sporadic inclusion body myositis. Sci. Transl. Med. 8331ra41. 10.1126/scitranslmed.aad4583, PMID: 27009270 PMC5043094

[ref3] Aizer A. Kafri P. Kalo A. Shav-Tal Y. (2013). The P body protein Dcp1a is hyper-phosphorylated during mitosis. PLoS One 8e49783. 10.1371/journal.pone.0049783, PMID: 23300942 PMC3534667

[ref4] Alberti S. (2017). Phase separation in biology. Curr. Biol. 27, R1097–R1102. 10.1016/j.cub.2017.08.069, PMID: 29065286

[ref5] Alberti S. Halfmann R. King O. Kapila A. Lindquist S. (2009). A systematic survey identifies prions and illuminates sequence features of prionogenic proteins. Cell 137, 146–158. 10.1016/j.cell.2009.02.044, PMID: 19345193 PMC2683788

[ref6] Alberti S. Mateju D. Mediani L. Carra S. (2017). Granulostasis: protein quality control of RNP granules. Front. Mol. Neurosci. 10:84. 10.3389/fnmol.2017.00084, PMID: 28396624 PMC5367262

[ref7] Allain F. H. Bouvet P. Dieckmann T. Feigon J. (2000). Molecular basis of sequence-specific recognition of pre-ribosomal RNA by nucleolin. EMBO J. 19, 6870–6881. 10.1093/emboj/19.24.6870, PMID: 11118222 PMC305906

[ref8] Andersen J. S. Lyon C. E. Fox A. H. Leung A. K. Lam Y. W. Steen H. (2002). Directed proteomic analysis of the human nucleolus. Curr. Biol. 12, 1–11. 10.1016/S0960-9822(01)00650-9 11790298

[ref9] Andrade L. E. Tan E. M. Chan E. K. (1993). Immunocytochemical analysis of the coiled body in the cell cycle and during cell proliferation. Proc. Natl. Acad. Sci. U. S. A. 90, 1947–1951. 8446613 10.1073/pnas.90.5.1947 PMC45997

[ref10] Arai T. Hasegawa M. Akiyama H. Ikeda K. Nonaka T. Mori H.. (2006). TDP-43 is a component of ubiquitin-positive tau-negative inclusions in frontotemporal lobar degeneration and amyotrophic lateral sclerosis. Biochem. Biophys. Res. Commun. 351, 602–611. 10.1016/j.bbrc.2006.10.093, PMID: 17084815

[ref11] Araya N. Hiraga H. Kako K. Arao Y. Kato S. Fukamizu A. (2005). Transcriptional down-regulation through nuclear exclusion of EWS methylated by PRMT1. Biochem. Biophys. Res. Commun. 329, 653–660. 10.1016/j.bbrc.2005.02.018, PMID: 15737635

[ref12] Arrigo A. P. Suhan J. P. Welch W. J. (1988). Dynamic changes in the structure and intracellular locale of the mammalian low-molecular-weight heat shock protein. Mol. Cell. Biol. 8, 5059–5071. 10.1128/MCB.8.12.5059, PMID: 3072471 PMC365607

[ref13] Banani S. F. Lee H. O. Hyman A. A. Rosen M. K. (2017). Biomolecular condensates: organizers of cellular biochemistry. Nat. Rev. Mol. Cell Biol. 18, 285–298. 10.1038/nrm.2017.7, PMID: 28225081 PMC7434221

[ref14] Banfi S. Servadio A. Chung M. Y. Kwiatkowski T. J. Jr. McCall A. E. Duvick L. A. (1994). Identification and characterization of the gene causing type 1 spinocerebellar ataxia. Nat. Genet. 7, 513–520. 10.1038/ng0894-513 7951322

[ref15] Barmada S. J. Serio A. Arjun A. Bilican B. Daub A. Ando D. M.. (2014). Autophagy induction enhances TDP43 turnover and survival in neuronal ALS models. Nat. Chem. Biol. 10, 677–685. 10.1038/nchembio.1563, PMID: 24974230 PMC4106236

[ref16] Becker L. A. Huang B. Bieri G. Ma R. Knowles D. A. Jafar-Nejad P.. (2017). Therapeutic reduction of ataxin-2 extends lifespan and reduces pathology in TDP-43 mice. Nature 544, 367–371. 10.1038/nature22038, PMID: 28405022 PMC5642042

[ref17] Bedford M. T. Clarke S. G. (2009). Protein arginine methylation in mammals: who, what, and why. Mol. Cell 33, 1–13. 10.1016/j.molcel.2008.12.013, PMID: 19150423 PMC3372459

[ref18] Bedford M. T. Frankel A. Yaffe M. B. Clarke S. Leder P. Richard S. (2000). Arginine methylation inhibits the binding of proline-rich ligands to Src homology 3, but not WW, domains. J. Biol. Chem. 275, 16030–16036. 10.1074/jbc.M909368199, PMID: 10748127

[ref19] Bedford M. T. Richard S. (2005). Arginine methylation an emerging regulator of protein function. Mol. Cell 18, 263–272. 10.1016/j.molcel.2005.04.003, PMID: 15866169

[ref20] Benatar M. Wuu J. Fernandez C. Weihl C. C. Katzen H. Steele J.. (2013). Motor neuron involvement in multisystem proteinopathy: implications for ALS. Neurology 80, 1874–1880. 10.1212/WNL.0b013e3182929fc3, PMID: 23635965 PMC3908355

[ref21] Bertolotti A. Lutz Y. Heard D. J. Chambon P. Tora L. (1996). hTAF(II)68, a novel RNA/ssDNA-binding protein with homology to the pro-oncoproteins TLS/FUS and EWS is associated with both TFIID and RNA polymerase II. EMBO J. 15, 5022–5031. 10.1002/j.1460-2075.1996.tb00882.x, PMID: 8890175 PMC452240

[ref22] Blackwell E. Ceman S. (2011). A new regulatory function of the region proximal to the RGG box in the fragile X mental retardation protein. J. Cell Sci. 124, 3060–3065. 10.1242/jcs.086751 21868366 PMC3172185

[ref23] Blackwell E. Zhang X. Ceman S. (2010). Arginines of the RGG box regulate FMRP association with polyribosomes and mRNA. Hum. Mol. Genet. 19, 1314–1323. 10.1093/hmg/ddq007, PMID: 20064924 PMC2838539

[ref24] Boeynaems S. Alberti S. Fawzi N. L. Mittag T. Polymenidou M. Rousseau F.. (2018). Protein phase separation: a new phase in cell biology. Trends Cell Biol. 28, 420–435. 10.1016/j.tcb.2018.02.004, PMID: 29602697 PMC6034118

[ref25] Boisvert F. M. van Koningsbruggen S. Navascués J. Lamond A. I. (2007). The multifunctional nucleolus. Nat. Rev. Mol. Cell Biol. 8, 574–585. 10.1038/nrm2184, PMID: 17519961

[ref26] Bouchard J. J. Otero J. H. Scott D. C. Szulc E. Martin E. W. Sabri N.. (2018). Cancer mutations of the tumor suppressor SPOP disrupt the formation of active, phase-separated compartments. Mol. Cell 72, 19–36.e18. 10.1016/j.molcel.2018.08.027, PMID: 30244836 PMC6179159

[ref27] Boulay G. Sandoval G. J. Riggi N. Iyer S. Buisson R. Naigles B.. (2017). Cancer-specific retargeting of BAF complexes by a prion-like domain. Cell 171, 163–178.e119. 10.1016/j.cell.2017.07.036, PMID: 28844694 PMC6791823

[ref28] Brangwynne C. P. Eckmann C. R. Courson D. S. Rybarska A. Hoege C. Gharakhani J.. (2009). Germline P granules are liquid droplets that localize by controlled dissolution/condensation. Science 324, 1729–1732. 10.1126/science.1172046, PMID: 19460965

[ref29] Brangwynne C. P. Mitchison T. J. Hyman A. A. (2011). Active liquid-like behavior of nucleoli determines their size and shape in *Xenopus laevis* oocytes. Proc. Natl. Acad. Sci. U. S. A. 108, 4334–4339. 10.1073/pnas.1017150108 21368180 PMC3060270

[ref30] Buratti E. Baralle F. E. (2008). Multiple roles of TDP-43 in gene expression, splicing regulation, and human disease. Front. Biosci. 13, 867–878. 10.2741/2727, PMID: 17981595

[ref31] Buratti E. Baralle F. E. (2010). The multiple roles of TDP-43 in pre-mRNA processing and gene expression regulation. RNA Biol. 7, 420–429. 10.4161/rna.7.4.12205 20639693

[ref32] Buratti E. Brindisi A. Giombi M. Tisminetzky S. Ayala Y. M. Baralle F. E. (2005). TDP-43 binds heterogeneous nuclear ribonucleoprotein A/B through its C-terminal tail: an important region for the inhibition of cystic fibrosis transmembrane conductance regulator exon 9 splicing. J. Biol. Chem. 280, 37572–37584. 10.1074/jbc.M505557200, PMID: 16157593

[ref33] Capponi S. Geuens T. Geroldi A. Origone P. Verdiani S. Cichero E.. (2016). Molecular chperones in the pathogenesis of amyotrophic lateral sclerosis: the role of HSPB1. Hum. Mutat. 37, 1202–1208. 10.1002/humu.23062, PMID: 27492805 PMC5108433

[ref34] Carmo-Fonseca M. Ferreira J. Lamond A. I. (1993). Assembly of snRNP-containing coiled bodies is regulated in interphase and mitosis–evidence that the coiled body is a kinetic nuclear structure. J. Cell Biol. 120, 841–852. 7679389 10.1083/jcb.120.4.841 PMC2200076

[ref35] Chen-Plotkin A. S. Lee V. M. Trojanowski J. Q. (2010). TAR DNA-binding protein 43 in neurodegenerative disease. Nat. Rev. Neurol. 6, 211–220. 10.1038/nrneurol.2010.18, PMID: 20234357 PMC2892118

[ref36] Clemson C. M. Hutchinson J. N. Sara S. A. Ensminger A. W. Fox A. H. Chess A.. (2009). An architectural role for a nuclear noncoding RNA: NEAT1 RNA is essential for the structure of paraspeckles. Mol. Cell 33, 717–726. 10.1016/j.molcel.2009.01.026, PMID: 19217333 PMC2696186

[ref37] Collier N. C. Schlesinger M. J. (1986). The dynamic state of heat shock proteins in chicken embryo fibroblasts. J. Cell Biol. 103, 1495–1507. 10.1083/jcb.103.4.1495, PMID: 3533955 PMC2114322

[ref38] Collins M. O. Yu L. Campuzano I. Grant S. G. Choudhary J. S. (2008). Phosphoproteomic analysis of the mouse brain cytosol reveals a predominance of protein phosphorylation in regions of intrinsic sequence disorder. Mol. Cell. Proteomics 7, 1331–1348. 10.1074/mcp.M700564-MCP200, PMID: 18388127

[ref39] Couthouis J. Hart M. P. Shorter J. DeJesus-Hernandez M. Erion R. Oristano R. (2011). A yeast functional screen predicts new candidate ALS disease genes. Proc. Natl. Acad. Sci. U. S. A. 108, 20881–20890. 10.1073/pnas.1109434108 22065782 PMC3248518

[ref40] Da Cruz S. Cleveland D. W. (2011). Understanding the role of TDP-43 and FUS/TLS in ALS and beyond. Curr. Opin. Neurobiol. 21, 904–919. 10.1016/j.conb.2011.05.029, PMID: 21813273 PMC3228892

[ref41] Dammer E. B. Fallini C. Gozal Y. M. Duong D. M. Rossoll W. Xu P.. (2012). Coaggregation of RNA-binding proteins in a model of TDP-43 proteinopathy with selective RGG motif methylation and a role for RRM1 ubiquitination. PLoS One 7e38658. 10.1371/journal.pone.0038658, PMID: 22761693 PMC3380899

[ref42] Drepper C. Sendtner M. (2011). A new postal code for dendritic mRNA transport in neurons. EMBO Rep. 12, 614–616. 10.1038/embor.2011.119, PMID: 21681203 PMC3128960

[ref43] Elden A. C. Kim H. J. Hart M. P. Chen-Plotkin A. S. Johnson B. S. Fang X.. (2010). Ataxin-2 intermediate-length polyglutamine expansions are associated with increased risk for ALS. Nature 466, 1069–1075. 10.1038/nature09320, PMID: 20740007 PMC2965417

[ref44] Eulalio A. Behm-Ansmant I. Schweizer D. Izaurralde E. (2007). P-body formation is a consequence, not the cause, of RNA-mediated gene silencing. Mol. Cell. Biol. 27, 3970–3981. 10.1128/MCB.00128-07, PMID: 17403906 PMC1900022

[ref45] Feric M. Vaidya N. Harmon T. S. Mitrea D. M. Zhu L. Richardson T. M.. (2016). Coexisting liquid phases underlie nucleolar subcompartments. Cell 165, 1686–1697. 10.1016/j.cell.2016.04.047, PMID: 27212236 PMC5127388

[ref46] Fidaleo M. De Paola E. Paronetto M. P. (2016). The RNA helicase A in malignant transformation. Oncotarget 7, 28711–28723. 10.18632/oncotarget.7377, PMID: 26885691 PMC5053757

[ref47] Fox A. H. Bond C. S. Lamond A. I. (2005). P54nrb forms a heterodimer with PSP1 that localizes to paraspeckles in an RNA-dependent manner. Mol. Biol. Cell 16, 5304–5315. 10.1091/mbc.E05-06-0587 16148043 PMC1266428

[ref48] Fox A. H. Lam Y. W. Leung A. K. Lyon C. E. Andersen J. Mann M.. (2002). Paraspeckles: a novel nuclear domain. Curr. Biol. 12, 13–25. 10.1016/S0960-9822(01)00632-7, PMID: 11790299

[ref49] Franks T. M. Lykke-Andersen J. (2008). The control of mRNA decapping and P-body formation. Mol. Cell 32, 605–615. 10.1016/j.molcel.2008.11.001, PMID: 19061636 PMC2630519

[ref50] Frey M. R. Matera A. G. (2001). RNA-mediated interaction of Cajal bodies and U2 snRNA genes. J. Cell Biol. 154, 499–509. 10.1083/jcb.200105084, PMID: 11489914 PMC2196410

[ref51] Gall J. G. Bellini M. Wu Z. Murphy C. (1999). Assembly of the nuclear transcription and processing machinery: Cajal bodies (coiled bodies) and transcriptosomes. Mol. Biol. Cell 10, 4385–4402. 10.1091/mbc.10.12.4385 10588665 PMC25765

[ref52] Ganassi M. Mateju D. Bigi I. Mediani L. Poser I. Lee H. O.. (2016). A surveillance function of the HSPB8-BAG3-HSP70 chaperone complex ensures stress granule integrity and dynamism. Mol. Cell 63, 796–810. 10.1016/j.molcel.2016.07.021, PMID: 27570075

[ref53] Gilks N. Kedersha N. Ayodele M. Shen L. Stoecklin G. Dember L. M. (2004). Stress granule assembly is mediated by prion-like aggregation of TIA-1. Mol. Biol. Cell 15, 5383–5398. 10.1091/mbc.e04-08-0715 15371533 PMC532018

[ref54] Guo L. Kim H. J. Wang H. Monaghan J. Freyermuth F. Sung J. C.. (2018). Nuclear-import receptors reverse aberrant phase transitions of RNA-binding proteins with prion-like domains. Cell 173, 677–692.e620. 10.1016/j.cell.2018.03.002, PMID: 29677512 PMC5911940

[ref55] Halfmann R. Alberti S. Krishnan R. Lyle N. O’Donnell C. W. King O. D.. (2011). Opposing effects of glutamine and asparagine govern prion formation by intrinsically disordered proteins. Mol. Cell 43, 72–84. 10.1016/j.molcel.2011.05.013, PMID: 21726811 PMC3132398

[ref56] Han T. W. Kato M. Xie S. Wu L. C. Mirzaei H. Pei J.. (2012). Cell-free formation of RNA granules: bound RNAs identify features and components of cellular assemblies. Cell 149, 768–779. 10.1016/j.cell.2012.04.016, PMID: 22579282

[ref57] Hanahan D. Weinberg R. A. (2011). Hallmarks of cancer: the next generation. Cell 144, 646–674. 10.1016/j.cell.2011.02.013, PMID: 21376230

[ref58] Handwerger K. E. Murphy C. Gall J. G. (2003). Steady-state dynamics of Cajal body components in the *Xenopus* germinal vesicle. J. Cell Biol. 160, 495–504. 10.1083/jcb.200212024, PMID: 12591912 PMC2173734

[ref59] Hennig S. Kong G. Mannen T. Sadowska A. Kobelke S. Blythe A.. (2015). Prion-like domains in RNA binding proteins are essential for building subnuclear paraspeckles. J. Cell Biol. 210, 529–539. 10.1083/jcb.201504117, PMID: 26283796 PMC4539981

[ref60] Hernandez-Verdun D. Roussel P. Gébrane-Younès J. (2002). Emerging concepts of nucleolar assembly. J. Cell Sci. 115, 2265–2270. PMID: 12006611 10.1242/jcs.115.11.2265

[ref61] Hnisz D. Shrinivas K. Young R. A. Chakraborty A. K. Sharp P. A. (2017). A phase separation model for transcriptional control. Cell 169, 13–23. 10.1016/j.cell.2017.02.007, PMID: 28340338 PMC5432200

[ref62] Hoell J. I. Larsson E. Runge S. Nusbaum J. D. Duggimpudi S. Farazi T. A.. (2011). RNA targets of wild-type and mutant FET family proteins. Nat. Struct. Mol. Biol. 18, 1428–1431. 10.1038/nsmb.2163, PMID: 22081015 PMC3230689

[ref63] Hyman A. A. Weber C. A. Jülicher F. (2014). Liquid-liquid phase separation in biology. Annu. Rev. Cell Dev. Biol. 30, 39–58. 10.1146/annurev-cellbio-100913-013325, PMID: 25288112

[ref64] Iakoucheva L. M. Radivojac P. Brown C. J. O’Connor T. R. Sikes J. G. Obradovic Z.. (2004). The importance of intrinsic disorder for protein phosphorylation. Nucleic Acids Res. 32, 1037–1049. 10.1093/nar/gkh253, PMID: 14960716 PMC373391

[ref65] Irwin S. Vandelft M. Pinchev D. Howell J. L. Graczyk J. Orr H. T. (2005). RNA association and nucleocytoplasmic shuttling by ataxin-1. J. Cell Sci. 118, 233–242. 10.1242/jcs.01611 15615787

[ref66] Jackrel M. E. DeSantis M. E. Martinez B. A. Castellano L. M. Stewart R. M. Caldwell K. A.. (2014). Potentiated Hsp104 variants antagonize diverse proteotoxic misfolding events. Cell 156, 170–182. 10.1016/j.cell.2013.11.047, PMID: 24439375 PMC3909490

[ref67] Jackrel M. E. Shorter J. (2014). Potentiated Hsp104 variants suppress toxicity of diverse neurodegenerative disease-linked proteins. Dis. Model. Mech. 7, 1175–1184. 10.1242/dmm.016113, PMID: 25062688 PMC4174528

[ref68] Jackrel M. E. Shorter J. (2015). Engineering enhanced protein disaggregases for neurodegenerative disease. Prion 9, 90–109. 10.1080/19336896.2015.1020277, PMID: 25738979 PMC4601286

[ref69] Jain A. Vale R. D. (2017). RNA phase transitions in repeat expansion disorders. Nature 546, 243–247. 10.1038/nature22386, PMID: 28562589 PMC5555642

[ref70] Jain S. Wheeler J. R. Walters R. W. Agrawal A. Barsic A. Parker R. (2016). ATPase-modulated stress granules contain a diverse proteome and substructure. Cell 164, 487–498. 10.1016/j.cell.2015.12.038, PMID: 26777405 PMC4733397

[ref71] Johansson H. O. Karlström G. Tjerneld F. Haynes C. A. (1998). Driving forces for phase separation and partitioning in aqueous two-phase systems. J. Chromatogr. B Biomed. Sci. Appl. 711, 3–17. 10.1016/S0378-4347(97)00585-9, PMID: 9699970

[ref72] Kaehler C. Isensee J. Nonhoff U. Terrey M. Hucho T. Lehrach H.. (2012). Ataxin-2-like is a regulator of stress granules and processing bodies. PLoS One 7e50134. 10.1371/journal.pone.0050134, PMID: 23209657 PMC3507954

[ref73] Kato M. Han T. W. Xie S. Shi K. Du X. Wu L. C.. (2012). Cell-free formation of RNA granules: low complexity sequence domains form dynamic fibers within hydrogels. Cell 149, 753–767. 10.1016/j.cell.2012.04.017, PMID: 22579281 PMC6347373

[ref74] Kedersha N. Anderson P. (2007). Mammalian stress granules and processing bodies. Methods Enzymol. 431, 61–81. 10.1016/S0076-6879(07)31005-7 17923231

[ref75] Kedersha N. L. Gupta M. Li W. Miller I. Anderson P. (1999). RNA-binding proteins TIA-1 and TIAR link the phosphorylation of eIF-2 alpha to the assembly of mammalian stress granules. J. Cell Biol. 147, 1431–1442. 10.1083/jcb.147.7.1431, PMID: 10613902 PMC2174242

[ref76] Kedersha N. Stoecklin G. Ayodele M. Yacono P. Lykke-Andersen J. Fritzler M. J.. (2005). Stress granules and processing bodies are dynamically linked sites of mRNP remodeling. J. Cell Biol. 169, 871–884. 10.1083/jcb.200502088, PMID: 15967811 PMC2171635

[ref77] Kieran D. Kalmar B. Dick J. R. Riddoch-Contreras J. Burnstock G. Greensmith L. (2004). Treatment with arimoclomol, a coinducer of heat shock proteins, delays disease progression in ALS mice. Nat. Med. 10, 402–405. 10.1038/nm1021, PMID: 15034571

[ref78] Kiledjian M. Dreyfuss G. (1992). Primary structure and binding activity of the hnRNP U protein: binding RNA through RGG box. EMBO J. 11, 2655–2664. 10.1002/j.1460-2075.1992.tb05331.x, PMID: 1628625 PMC556741

[ref79] Kim H. J. Kim N. C. Wang Y. D. Scarborough E. A. Moore J. Diaz Z.. (2013). Mutations in prion-like domains in hnRNPA2B1 and hnRNPA1 cause multisystem proteinopathy and ALS. Nature 495, 467–473. 10.1038/nature11922, PMID: 23455423 PMC3756911

[ref80] King O. D. Gitler A. D. Shorter J. (2012). The tip of the iceberg: RNA-binding proteins with prion-like domains in neurodegenerative disease. Brain Res. 1462, 61–80. 10.1016/j.brainres.2012.01.016, PMID: 22445064 PMC3372647

[ref81] Koritzinsky M. Magagnin M. G. van den Beucken T. Seigneuric R. Savelkouls K. Dostie J.. (2006). Gene expression during acute and prolonged hypoxia is regulated by distinct mechanisms of translational control. EMBO J. 25, 1114–1125. 10.1038/sj.emboj.7600998, PMID: 16467844 PMC1409715

[ref82] Kwiatkowski T. J. Bosco D. A. Leclerc A. L. Tamrazian E. Vanderburg C. R. Russ C.. (2009). Mutations in the FUS/TLS gene on chromosome 16 cause familial amyotrophic lateral sclerosis. Science 323, 1205–1208. 10.1126/science.1166066, PMID: 19251627

[ref83] Le Ber I. Van Bortel I. Nicolas G. Bouya-Ahmed K. Camuzat A. Wallon D. (2014). hnRNPA2B1 and hnRNPA1 mutations are rare in patients with “multisystem proteinopathy” and frontotemporal lobar degeneration phenotypes. Neurobiol. Aging 35934. 10.1016/j.neurobiolaging.2013.09.016 24119545

[ref84] Lee M. Sadowska A. Bekere I. Ho D. Gully B. S. Lu Y.. (2015). The structure of human SFPQ reveals a coiled-coil mediated polymer essential for functional aggregation in gene regulation. Nucleic Acids Res. 43, 3826–3840. 10.1093/nar/gkv156, PMID: 25765647 PMC4402515

[ref85] Li P. Banjade S. Cheng H. C. Kim S. Chen B. Guo L.. (2012). Phase transitions in the assembly of multivalent signalling proteins. Nature 483, 336–340. 10.1038/nature10879, PMID: 22398450 PMC3343696

[ref86] Li G. Ci W. Karmakar S. Chen K. Dhar R. Fan Z.. (2014). SPOP promotes tumorigenesis by acting as a key regulatory hub in kidney cancer. Cancer Cell 25, 455–468. 10.1016/j.ccr.2014.02.007, PMID: 24656772 PMC4443692

[ref87] Li Y. R. King O. D. Shorter J. Gitler A. D. (2013). Stress granules as crucibles of ALS pathogenesis. J. Cell Biol. 201, 361–372. 10.1083/jcb.201302044, PMID: 23629963 PMC3639398

[ref88] Li K. K. Lee K. A. (2000). Transcriptional activation by the Ewing’s sarcoma (EWS) oncogene can be cis-repressed by the EWS RNA-binding domain. J. Biol. Chem. 275, 23053–23058. 10.1074/jbc.M002961200, PMID: 10767297

[ref89] Li L. Lindquist S. (2000). Creating a protein-based element of inheritance. Science 287, 661–664. 10.1126/science.287.5453.661, PMID: 10650001

[ref90] Lin Y. Currie S. L. Rosen M. K. (2017). Intrinsically disordered sequences enable modulation of protein phase separation through distributed tyrosine motifs. J. Biol. Chem. 292, 19110–19120. 10.1074/jbc.M117.800466, PMID: 28924037 PMC5704491

[ref91] Ling S. C. Polymenidou M. Cleveland D. W. (2013). Converging mechanisms in ALS and FTD: disrupted RNA and protein homeostasis. Neuron 79, 416–438. 10.1016/j.neuron.2013.07.033, PMID: 23931993 PMC4411085

[ref92] Lorenzetti D. Bohlega S. Zoghbi H. Y. (1997). The expansion of the CAG repeat in ataxin-2 is a frequent cause of autosomal dominant spinocerebellar ataxia. Neurology 49, 1009–1013. 10.1212/WNL.49.4.1009, PMID: 9339681

[ref93] Machyna M. Heyn P. Neugebauer K. M. (2013). Cajal bodies: where form meets function. Wiley Interdiscip. Rev. RNA 4, 17–34. 10.1002/wrna.1139, PMID: 23042601

[ref94] Machyna M. Kehr S. Straube K. Kappei D. Buchholz F. Butter F.. (2014). The coilin interactome identifies hundreds of small noncoding RNAs that traffic through Cajal bodies. Mol. Cell 56, 389–399. 10.1016/j.molcel.2014.10.004, PMID: 25514182

[ref95] Mackenzie I. R. Rademakers R. Neumann M. (2010). TDP-43 and FUS in amyotrophic lateral sclerosis and frontotemporal dementia. Lancet Neurol. 9, 995–1007. 10.1016/S1474-4422(10)70195-2, PMID: 20864052

[ref96] March Z. M. King O. D. Shorter J. (2016). Prion-like domains as epigenetic regulators, scaffolds for subcellular organization, and drivers of neurodegenerative disease. Brain Res. 1647, 9–18. 10.1016/j.brainres.2016.02.037, PMID: 26996412 PMC5003744

[ref97] Marzahn M. R. Marada S. Lee J. Nourse A. Kenrick S. Zhao H.. (2016). Higher-order oligomerization promotes localization of SPOP to liquid nuclear speckles. EMBO J. 35, 1254–1275. 10.15252/embj.201593169, PMID: 27220849 PMC4910529

[ref98] Masison D. C. Maddelein M. L. Wickner R. B. (1997). The prion model for [URE3] of yeast: spontaneous generation and requirements for propagation. Proc. Natl. Acad. Sci. U. S. A. 94, 12503–12508. 9356479 10.1073/pnas.94.23.12503 PMC25018

[ref99] Mateju D. Franzmann T. M. Patel A. Kopach A. Boczek E. E. Maharana S.. (2017). An aberrant phase transition of stress granules triggered by misfolded protein and prevented by chaperone function. EMBO J. 36, 1669–1687. 10.15252/embj.201695957, PMID: 28377462 PMC5470046

[ref100] Matera A. G. (1999). Nuclear bodies: multifaceted subdomains of the interchromatin space. Trends Cell Biol. 9, 302–309. 10.1016/S0962-8924(99)01606-2, PMID: 10407409

[ref101] Mazroui R. Huot M. E. Tremblay S. Boilard N. Labelle Y. Khandjian E. W. (2003). Fragile X mental retardation protein determinants required for its association with polyribosomal mRNPs. Hum. Mol. Genet. 12, 3087–3096. 10.1093/hmg/ddg335, PMID: 14532325

[ref102] Mokas S. Mills J. R. Garreau C. Fournier M. J. Robert F. Arya P. (2009). Uncoupling stress granule assembly and translation initiation inhibition. Mol. Biol. Cell 20, 2673–2683. 10.1091/mbc.e08-10-1061 19369421 PMC2688547

[ref103] Molliex A. Temirov J. Lee J. Coughlin M. Kanagaraj A. P. Kim H. J.. (2015). Phase separation by low complexity domains promotes stress granule assembly and drives pathological fibrillization. Cell 163, 123–133. 10.1016/j.cell.2015.09.015, PMID: 26406374 PMC5149108

[ref104] Moutaoufik M. T. El Fatimy R. Nassour H. Gareau C. Lang J. Tanguay R. M.. (2014). UVC-induced stress granules in mammalian cells. PLoS One 9:e112742. 10.1371/journal.pone.0112742, PMID: 25409157 PMC4237350

[ref105] Munder M. C. Midtvedt D. Franzmann T. Nüske E. Otto O. Herbig M. (2016). A pH-driven transition of the cytoplasm from a fluid- to a solid-like state promotes entry into dormancy. elife 5e09347. 10.7554/eLife.09347 PMC4850707 27003292

[ref106] Murakami T. Qamar S. Lin J. Q. Schierle G. S. Rees E. Miyashita A.. (2015). ALS/FTD mutation-induced phase transition of FUS liquid droplets and reversible hydrogels into irreversible hydrogels impairs RNP granule function. Neuron 88, 678–690. 10.1016/j.neuron.2015.10.030, PMID: 26526393 PMC4660210

[ref107] Nagai Y. Kojima T. Muro Y. Hachiya T. Nishizawa Y. Wakabayashi T.. (1997). Identification of a novel nuclear speckle-type protein, SPOP. FEBS Lett. 418, 23–26. 10.1016/S0014-5793(97)01340-9, PMID: 9414087

[ref108] Neumann M. Bentmann E. Dormann D. Jawaid A. DeJesus-Hernandez M. Ansorge O.. (2011). FET proteins TAF15 and EWS are selective markers that distinguish FTLD with FUS pathology from amyotrophic lateral sclerosis with FUS mutations. Brain 134, 2595–2609. 10.1093/brain/awr201, PMID: 21856723 PMC3170539

[ref109] Neumann M. Rademakers R. Roeber S. Baker M. Kretzschmar H. A. Mackenzie I. R. (2009). A new subtype of frontotemporal lobar degeneration with FUS pathology. Brain 132, 2922–2931. 10.1093/brain/awp214, PMID: 19674978 PMC2768659

[ref110] Neumann M. Sampathu D. M. Kwong L. K. Truax A. C. Micsenyi M. C. Chou T. T.. (2006). Ubiquitinated TDP-43 in frontotemporal lobar degeneration and amyotrophic lateral sclerosis. Science 314, 130–133. 10.1126/science.1134108, PMID: 17023659

[ref111] Nott T. J. Petsalaki E. Farber P. Jervis D. Fussner E. Plochowietz A.. (2015). Phase transition of a disordered nuage protein generates environmentally responsive membraneless organelles. Mol. Cell 57, 936–947. 10.1016/j.molcel.2015.01.013, PMID: 25747659 PMC4352761

[ref112] Ohn T. Kedersha N. Hickman T. Tisdale S. Anderson P. (2008). A functional RNAi screen links O-GlcNAc modification of ribosomal proteins to stress granule and processing body assembly. Nat. Cell Biol. 10, 1224–1231. 10.1038/ncb1783, PMID: 18794846 PMC4318256

[ref113] Orr H. T. (2012). Cell biology of spinocerebellar ataxia. J. Cell Biol. 197, 167–177. 10.1083/jcb.201105092, PMID: 22508507 PMC3328388

[ref114] Panas M. D. Ivanov P. Anderson P. (2016). Mechanistic insights into mammalian stress granule dynamics. J. Cell Biol. 215, 313–323. 10.1083/jcb.201609081, PMID: 27821493 PMC5100297

[ref115] Parker R. Sheth U. (2007). P bodies and the control of mRNA translation and degradation. Mol. Cell 25, 635–646. 10.1016/j.molcel.2007.02.011 17349952

[ref116] Paronetto M. P. (2013). Ewing sarcoma protein: a key player in human cancer. Int. J. Cell Biol. 2013642853. 10.1155/2013/642853, PMID: 24082883 PMC3776376

[ref117] Paronetto M. P. Miñana B. Valcárcel J. (2011). The Ewing sarcoma protein regulates DNA damage-induced alternative splicing. Mol. Cell 43, 353–368. 10.1016/j.molcel.2011.05.035, PMID: 21816343

[ref118] Polo S. E. Jackson S. P. (2011). Dynamics of DNA damage response proteins at DNA breaks: a focus on protein modifications. Genes Dev. 25, 409–433. 10.1101/gad.2021311, PMID: 21363960 PMC3049283

[ref119] Pothof J. Verkaik N. S. van IJcken W. Wiemer E. A. Ta V. T. van der Horst G. T.. (2009). MicroRNA-mediated gene silencing modulates the UV-induced DNA-damage response. EMBO J. 28, 2090–2099. 10.1038/emboj.2009.156, PMID: 19536137 PMC2718280

[ref120] Protter D. S. W. Parker R. (2016). Principles and Properties of Stress Granules. Trends Cell Biol. 26, 668–679. 10.1016/j.tcb.2016.05.004, PMID: 27289443 PMC4993645

[ref121] Rai A. K. Chen J. X. Selbach M. Pelkmans L. (2018). Kinase-controlled phase transition of membraneless organelles in mitosis. Nature 559, 211–216. 10.1038/s41586-018-0279-8, PMID: 29973724

[ref122] Ramaswami M. Taylor J. P. Parker R. (2013). Altered ribostasis: RNA-protein granules in degenerative disorders. Cell 154, 727–736. 10.1016/j.cell.2013.07.038, PMID: 23953108 PMC3811119

[ref123] Reineke L. C. Tsai W. C. Jain A. Kaelber J. T. Jung S. Y. Lloyd R. E. (2017). Casein kinase 2 is linked to stress granule dynamics through phosphorylation of the stress granule nucleating protein G3BP1. Mol. Cell. Biol. 37, e00596–e00516. 10.1128/MCB.00596-16 27920254 PMC5288577

[ref124] Rhoads S. N. Monahan Z. T. Yee D. S. Shewmaker F. P. (2018). The role of post-translational modifications on prion-like aggregation and liquid-phase separation of FUS. Int. J. Mol. Sci. 19886. 10.3390/ijms19030886, PMID: 29547565 PMC5877747

[ref125] Robberecht W. Philips T. (2013). The changing scene of amyotrophic lateral sclerosis. Nat. Rev. Neurosci. 14, 248–264. 10.1038/nrn3430, PMID: 23463272

[ref126] Romero P. Obradovic Z. Li X. Garner E. C. Brown C. J. Dunker A. K. (2001). Sequence complexity of disordered protein. Proteins 42, 38–48. 10.1002/1097-0134(20010101)42:1<38::AID-PROT50>3.0.CO;2-3, PMID: 11093259

[ref127] Rusmini P. Cristofani R. Galbiati M. Cicardi M. E. Meroni M. Ferrari V. (2017). The role of the heat shock protein B8 (HSPB8) in motoneuron diseases. Front. Mol. Neurosci. 10176. 10.3389/fnmol.2017.00176 PMC5478700 28680390

[ref128] Sabari B. R. Dall’Agnese A. Boija A. Klein I. A. Coffey E. L. Shrinivas K.. (2018). Coactivator condensation at super-enhancers links phase separation and gene control. Science 361eaar3958. 10.1126/science.aar3958, PMID: 29930091 PMC6092193

[ref130] Schoch K. M. Miller T. M. (2017). Antisense oligonucleotides: translation from mouse models to human neurodegenerative diseases. Neuron 94, 1056–1070. 10.1016/j.neuron.2017.04.010, PMID: 28641106 PMC5821515

[ref131] Servadio A. Koshy B. Armstrong D. Antalffy B. Orr H. T. Zoghbi H. Y. (1995). Expression analysis of the ataxin-1 protein in tissues from normal and spinocerebellar ataxia type 1 individuals. Nat. Genet. 10, 94–98. 10.1083/jcb.201105092, PMID: 7647801

[ref132] Shaw P. J. Jordan E. G. (1995). The nucleolus. Annu. Rev. Cell Dev. Biol. 11, 93–121. 10.1146/annurev.cb.11.110195.000521, PMID: 8689574

[ref133] Shelkovnikova T. A. Kukharsky M. S. An H. Dimasi P. Alexeeva S. Shabir O. (2018). Protective paraspeckle hyper-assembly downstream of TDP-43 loss of function in amyotrophic lateral sclerosis. Mol. Neurodegener. 1330. 10.1186/s13024-018-0263-7 PMC5984788 29859124

[ref134] Sheth U. Parker R. (2003). Decapping and decay of messenger RNA occur in cytoplasmic processing bodies. Science 300, 805–808. 10.1126/science.1082320, PMID: 12730603 PMC1876714

[ref135] Shin Y. Brangwynne C. P. (2017). Liquid phase condensation in cell physiology and disease. Science 357eaaf4382. 10.1126/science.aaf4382, PMID: 28935776

[ref136] Shorter J. (2008). Hsp104: a weapon to combat diverse neurodegenerative disorders. Neurosignals 16, 63–74. 10.1159/000109760 18097161

[ref137] Shorter J. Lindquist S. (2005). Prions as adaptive conduits of memory and inheritance. Nat. Rev. Genet. 6, 435–450. 10.1038/nrg1616, PMID: 15931169

[ref138] Souquere S. Beauclair G. Harper F. Fox A. Pierron G. (2010). Highly ordered spatial organization of the structural long noncoding NEAT1 RNAs within paraspeckle nuclear bodies. Mol. Biol. Cell 21, 4020–4027. 10.1091/mbc.E10-08-0690 20881053 PMC2982136

[ref139] Souquere S. Mollet S. Kress M. Dautry F. Pierron G. Weil D. (2009). Unravelling the ultrastructure of stress granules and associated P-bodies in human cells. J. Cell Sci. 122, 3619–3626. 10.1242/jcs.054437 19812307

[ref140] Stetler A. Winograd C. Sayegh J. Cheever A. Patton E. Zhang X.. (2006). Identification and characterization of the methyl arginines in the fragile X mental retardation protein Fmrp. Hum. Mol. Genet. 15, 87–96. 10.1093/hmg/ddi429, PMID: 16319129

[ref141] Strome S. Wood W. B. (1983). Generation of asymmetry and segregation of germ-line granules in early *C. elegans* embryos. Cell 35, 15–25. 10.1016/0092-8674(83)90203-9, PMID: 6684994

[ref142] Svetoni F. Frisone P. Paronetto M. P. (2016). Role of FET proteins in neurodegenerative disorders. RNA Biol. 13, 1089–1102. 10.1080/15476286.2016.1211225, PMID: 27415968 PMC5100351

[ref143] Teixeira D. Sheth U. Valencia-Sanchez M. A. Brengues M. Parker R. (2005). Processing bodies require RNA for assembly and contain nontranslating mRNAs. RNA 11, 371–382. 10.1261/rna.7258505, PMID: 15703442 PMC1370727

[ref144] Ticozzi N. Vance C. Leclerc A. L. Keagle P. Glass J. D. McKenna-Yasek D.. (2011). Mutational analysis reveals the FUS homolog TAF15 as a candidate gene for familial amyotrophic lateral sclerosis. Am. J. Med. Genet. B Neuropsychiatr. Genet. 156B, 285–290. 10.1002/ajmg.b.31158, PMID: 21438137

[ref145] Toretsky J. A. Wright P. E. (2014). Assemblages: functional units formed by cellular phase separation. J. Cell Biol. 206, 579–588. 10.1083/jcb.201404124, PMID: 25179628 PMC4151146

[ref146] Torrente M. P. Shorter J. (2013). The metazoan protein disaggregase and amyloid depolymerase system: Hsp110, Hsp70, Hsp40, and small heat shock proteins. Prion 7, 457–463. 10.4161/pri.27531 24401655 PMC4201613

[ref147] Tourrière H. Chebli K. Zekri L. Courselaud B. Blanchard J. M. Bertrand E.. (2003). The RasGAP-associated endoribonuclease G3BP assembles stress granules. J. Cell Biol. 160, 823–831. 10.1083/jcb.200212128, PMID: 12642610 PMC2173781

[ref148] Tradewell M. L. Yu Z. Tibshirani M. Boulanger M. C. Durham H. D. Richard S. (2012). Arginine methylation by PRMT1 regulates nuclear-cytoplasmic localization and toxicity of FUS/TLS harbouring ALS-linked mutations. Hum. Mol. Genet. 21, 136–149. 10.1093/hmg/ddr448, PMID: 21965298

[ref149] Van Treeck B. Protter D. S. W. Matheny T. Khong A. Link C. D. Parker R. (2018). RNA self-assembly contributes to stress granule formation and defining the stress granule transcriptome. Proc. Natl. Acad. Sci. U. S. A. 115, 2734–2739. 10.1073/pnas.1800038115, PMID: 29483269 PMC5856561

[ref150] Vance C. Rogelj B. Hortobágyi T. De Vos K. J. Nishimura A. L. Sreedharan J.. (2009). Mutations in FUS, an RNA processing protein, cause familial amyotrophic lateral sclerosis type 6. Science 323, 1208–1211. 10.1126/science.1165942, PMID: 19251628 PMC4516382

[ref151] Vernon R. M. Chong P. A. Tsang B. Kim T. H. Bah A. Farber P. (2018). Pi-Pi contacts are an overlooked protein feature relevant to phase separation. elife 7e31486. 10.7554/eLife.31486 PMC5847340 29424691

[ref550] Vigh L. Literati P. N. Horvath I. TörÖk Z. Balogh G. Glatz A.. (1997). Bimoclomol: a nontoxic, hydroxylamine derivative wiht stress protein-inducing activity and cytoprotective effects. Nat. Med. 3, 1150–1154. 10.1038/nm1097-1150, PMID: 9334730

[ref152] Voronina E. Seydoux G. Sassone-Corsi P. Nagamori I. (2011). RNA granules in germ cells. Cold Spring Harb. Perspect. Biol. 3a002774. 10.1101/cshperspect.a002774, PMID: 21768607 PMC3225947

[ref153] West J. A. Mito M. Kurosaka S. Takumi T. Tanegashima C. Chujo T.. (2016). Structural, super-resolution microscopy analysis of paraspeckle nuclear body organization. J. Cell Biol. 214, 817–830. 10.1083/jcb.201601071, PMID: 27646274 PMC5037409

[ref154] White J. P. Lloyd R. E. (2012). Regulation of stress granules in virus systems. Trends Microbiol. 20, 175–183. 10.1016/j.tim.2012.02.001, PMID: 22405519 PMC3322245

[ref155] Whyte W. A. Orlando D. A. Hnisz D. Abraham B. J. Lin C. Y. Kagey M. H. (2013). Master transcription factors and mediator establish super-enhancers at key cell identity genes. Cell 153, 307–319. 10.1016/j.cell.2013.03.035 23582322 PMC3653129

[ref5555] Wils H. Kleinberger G. Janssens J. Pereson S. Joris C. Cuijt I. (2010). TDP-43 transgenic mice develop spastic paralysis and neuronal inclusions characteristic of ALS and frontotemporal lobar degeneration. Proc. Natl. Acad. Sci. USA: 107, 3858–3863. 10.1073/pnas.0912417107, PMID: 20133711 PMC2840518

[ref156] Wippich F. Bodenmiller B. Trajkovska M. G. Wanka S. Aebersold R. Pelkmans L. (2013). Dual specificity kinase DYRK3 couples stress granule condensation/dissolution to mTORC1 signaling. Cell 152, 791–805. 10.1016/j.cell.2013.01.033, PMID: 23415227

[ref157] Xiang S. Kato M. Wu L. C. Lin Y. Ding M. Zhang Y.. (2015). The LC domain of hnRNPA2 adopts similar conformations in hydrogel polymers, liquid-like droplets, and nuclei. Cell 163, 829–839. 10.1016/j.cell.2015.10.040, PMID: 26544936 PMC4879888

[ref158] Yasuda K. Clatterbuck-Soper S. F. Jackrel M. E. Shorter J. Mili S. (2017). FUS inclusions disrupt RNA localization by sequestering kinesin-1 and inhibiting microtubule detyrosination. J. Cell Biol. 216, 1015–1034. 10.1083/jcb.201608022, PMID: 28298410 PMC5379945

[ref159] Zinszner H. Sok J. Immanuel D. Yin Y. Ron D. (1997). TLS (FUS) binds RNA in vivo and engages in nucleo-cytoplasmic shuttling. J. Cell Sci. 110, 1741–1750. 9264461 10.1242/jcs.110.15.1741

